# Towards a complete structure of the hERG channel

**DOI:** 10.1186/1758-2946-5-S1-P7

**Published:** 2013-03-22

**Authors:** Peter Schmidtke, Ciantar Marine, Isabelle Theret, Pierre Ducrot

**Affiliations:** 1 Institut de Recherche Servier, Croissy-sur-Seine, 78920, France

## 

A lot of attention has been drawn to the voltage gated potassium channel Kv11.1 during the last decades. In the past, both, ligand and structure based methods intended to predict if a small molecule could cause fatal heart arrhythmias, "torsades de pointe" and sudden death. However, despite the wide interest for hERG, still no experimental 3D structure is available and therefore homology modelling of parts of the channel (generally only the pore domain) is currently used to gain structural insights [1].

Here a novel structural model of hERG is presented encompassing the full transmembrane segment of hERG, including the conduction pore and the voltage sensitive domain. Furthermore, cytoplasmic domains like the cyclic nucleotide binding domain and the PAS domain have been positioned in the overall structure. Subsequent molecular dynamics simulations allow gaining novel structural and dynamic insights into hERG functioning and perturbation.
